# Minimal access excision of aortic valve fibroelastoma: a case report and review of the literature

**DOI:** 10.1186/1749-8090-7-80

**Published:** 2012-09-03

**Authors:** Leanne Harling, Thanos Athanasiou, Hutan Ashrafian, John Kokotsakis, Virginia Brown, Anthony Nathan, Roberto Casula

**Affiliations:** 1 Department of Surgery and Cancer, Imperial College Healthcare NHS Trust, Imperial College London, 10th Floor, QEQM Building, St. Mary's Campus, Praed Street, London, W2 1NY, UK; 2 Department of Cardiology, Watford General Hospital, Vicarage Road, Herts, Watford, WD18 0HB, Ireland

**Keywords:** Fibroelastoma, Minimally invasive, Aortic valve

## Abstract

Papillary fibroelastomas are rare primary tumours of cardiac origin accounting for approximately 10% of all primary cardiac neoplasms. Due to a high thromboembolic risk, surgical excision is the mainstay of treatment in these patients and median sternotomy the most widely used approach. We describe the case of a 43 year-old lady presenting with acute myocardial infarction secondary to aortic valve papillary fibroelastoma subsequently excised using a minimal access technique. From our experience mini-sternotomy offers excellent exposure and allows for safe resection in such cases, improving cosmesis without compromising either intra or post-operative outcome.

## Background

Papillary fibroelastomas (PFE) are rare primary tumours of cardiac origin accounting for approximately 10% of all primary cardiac neoplasms [1]. They may occur anywhere on the endocardium, although are more common in the left heart and display a predilection for valvular structures. The aortic valve is the most commonly affected, with a number of such tumours arising from the non-coronary leaflet [2,3]. Whilst the majority are incidental findings on echocardiography, symptoms most commonly occur subsequent to embolisation, and may give rise to a wide variety of presenting features including neurological events (transient ischaemic attack, stroke, amaurosis fugax, spinal cord infarction) [4-6], acute coronary syndrome [7,8], and distal thromboembolism [9]. More rarely, cardiac conduction abnormalities, direct coronary ostial occlusion and outflow tract obstruction (in ventricular PFE) have been reported [10,11].

Macroscopically, papillary fibroelastomas are characterised by multiple frond-like fibrous projections creating a ‘sea anemone’ appearance when immersed in saline. Histologically, the tumour is comprised of an avascular fibroelastic core, made up of a hyalinised collagen matrix with a rim of smooth muscle cells and elastic fibres, lined by endocardial endothelium [12,13]. Immunohistochemical characteristics include positive staining for factor VIII-related antigen, CD34 and Vimentin confirming a vascular endothelial cell lining, and multilayered type IV collagen elastic proliferation deep to the surface membrane [14].

The aetiology of PFE remains largely unknown [3]. Recent hypotheses include fibroblast infiltration with organisation of mural thrombi [15], viral induced tumour growth [13], and an endothelial response to cardiac surgery, mechanical trauma or thoracic radiation [16,17]. Historically, PFE has also been described as both a true neoplasm and a hamartomatous lesion [18], whereas some authors have suggested they are merely an overgrowth of Lambl’s excrescences [19].

Over recent years, there has been an increase in the number of reports of minimally invasive, thoracoscopic and even robotic resections of aortic valve PFE (Table 1) [20-25]. A total of 9 cases have been reported to date, of which 7 describe PFE of the AV (5 non-coronary leaflet, 2 right coronary leaflet), 1 of the right atrium and 1 of the left ventricle. Both mini-sternotomy and mini-thoracotomy approaches (± thoracoscopic assistance) have been used with incision size ranging from 4 to 9 cm, although it seems that mini-sternotomy offers improved intra-operative exposure, reduced post-operative pain and less mammary scaring when compared to mini-thoracotomy in cases of AV PFE [26]. Whilst no long term follow up has been documented, short term results with such procedures are encouraging with a mean hospital stay of 4 ± 0.57 days and no adverse events reported prior to discharge. 

**Table 1:** Summary of published results

**Reference**	**n**	**Age (yrs)**	**M:F**	**Presentation**	**Site of lesion**	**Size of lesion (cm)**	**Access Route**	**Temp (°C)**	**CPB Time (mins)**	**X-Clamp Time (mins)**	**Outcome**	**Follow-up (Months)**
Zebele 2010 [25]	1	-	0:1	Not described	Non-coronary leaflet	1.0 × 1.0	7 cm skin incision with mini-sternotomy	-	-	-	Not described	-
Hsu 2006 [21]	4	54 ± 5	0:4	2 Embolic stroke, 1 sequential TIAs, and 1 SOBOE & lightheadedness	3 Non coronary leaflet 1 R coronary cusp	0.8 ± 0.3 × 0.65 ± 0.35	Partial sternotomy extended into 3rd ICS. Incision length 6.1 ± 1.4 cm		66 ± 7.5	34.5 ± 3	LOS 4 ± 0.5 days No complications until point of discharge	-
Je 2008 [22]	1	39	1:0	Asymptomatic. Incidental finding on investigation for hypertrophic cardiomyopathy	Medial side of antero-lateral papillary muscle of the LV	1.5 × 1.0	5 cm R anterior thoracotomy (camera control: AESOP 3000)	-	57	24	Uneventful recovery. Follow up echo no residual mass, normal MV	-
							Cannulation: RCFA, RCFV and RIJV.					
Grande 2007 [20]	1	22	1:0	Asymptomatic. Incidental finding on `TTE for MV prolapse	Ventricular aspect of R coronary leaflet	0.6 × 0.9	9 cm ‘J’ incision on midline from 3 cm below jugular notch to 2^nd^ ICS. Sternum opened from manubrium to 3rd ICS.	-	-	50	Discharge day 5. No complications at last follow up.	6
							Cannulation: Aorta, Right atrial appendage.					
Woo 2005 [24]	1	50	1:0	Asymptomatic, surveillance revealed non-specific T wave changes. TTE finding of PFE	Non-coronary leaflet	Diameter 1.0	Robotic: 5 cm right anterior mini-thoracotomy 2nd ICS.	30	-	48	Discharge day 3. No complications at last follow up.	1
							Robotic arms via stab incisions R 1st and 3rd ICS					
							Cannulation: RCFA, RCFV					
Kim 2007 [23]	1	62	1:0	Asymptomatic, surveillance CT following total laryngectomy for laryngeal cancer.	Right Atrium	-	4 cm R anterior mini-thoracotomy in 4th ICS	-	24	-	Discharge day 4. No complications until point of discharge.	-
							Cannulation: RCFA, RCFV					

TIA – Transient Ischaemic Attack; SOBOE – Shortness of Breath on Exertion; TTE – Trans Thoracic Echo; MV – Mitral Valve; LV – Left Ventricle; CT – Computerised Tomography; RCFA – Right Common Femoral Artery; RCFV – Right Common Femoral Vein; RIJV – Right Internal Jugular Vein; ICS – Inter-costal Space; LOS – length of Stay; CPB – Cardiopulmonary Bypass.

In this report we review the current literature on minimally invasive intervention for PFE and present a case of aortic valve PFE resected via mini-sternotomy.

## Case report

**Figure 1:**
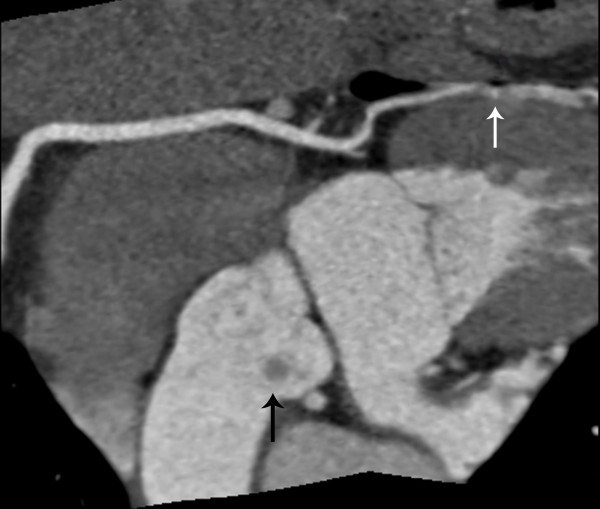
Computerised Tomography image demonstrating both an aortic valve lesion (black arrow) and an embolus to the distal left anterior descending artery (white arrow).

**Figure 2:**
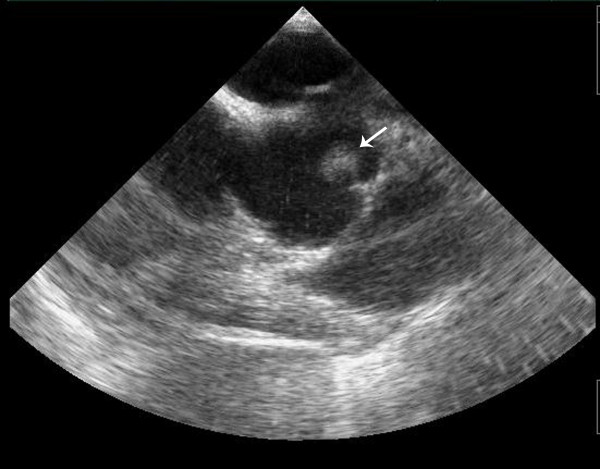
Trans-thoracic echocardiography demonstrating mobile mass on the left coronary cusp of the aortic valve.

**Figure 3:**
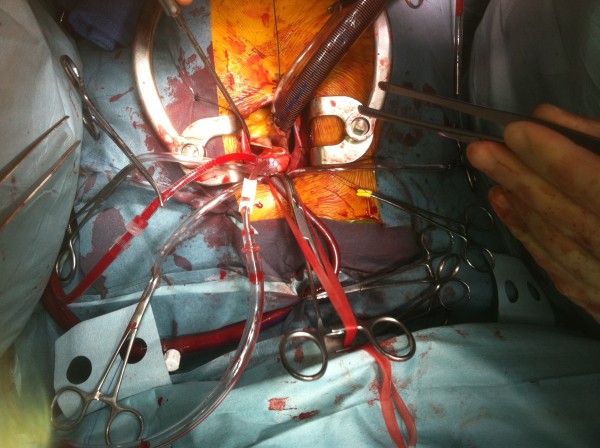
Minimal access approach and set-up.

**Figure 4:**
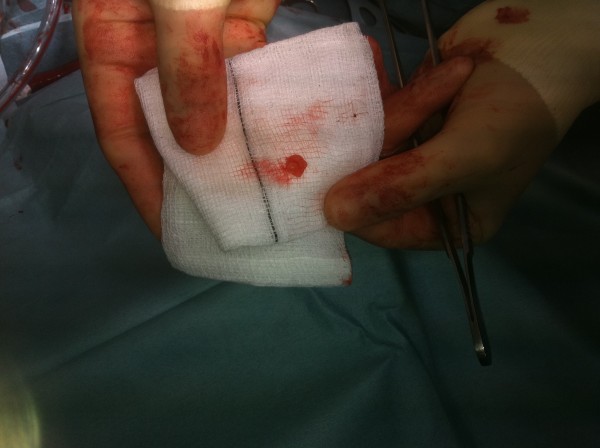
Excised fibroelastoma.

**Figure 5:**
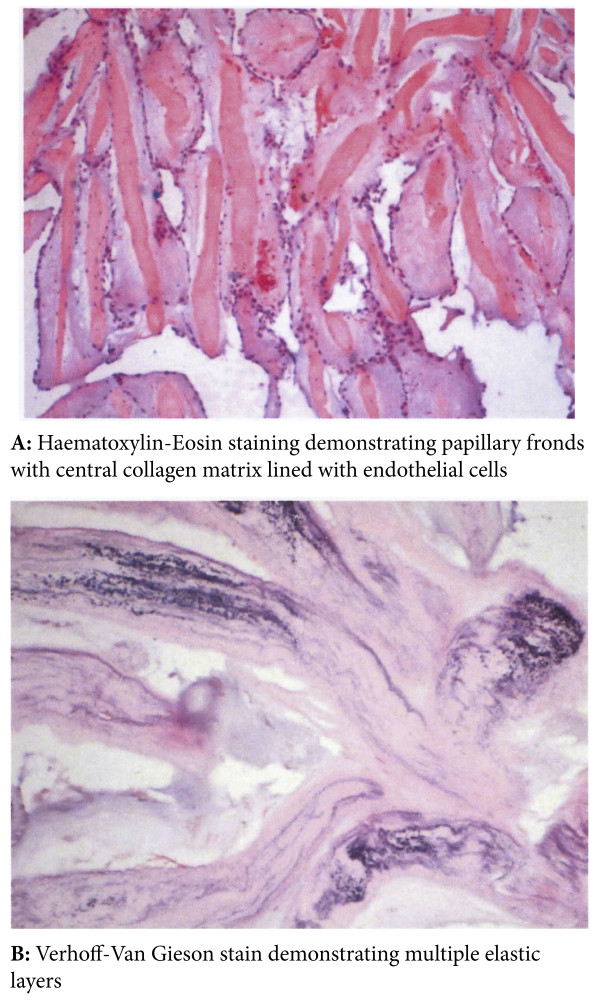
Histology of the excised PFE.

A 43-year-old lady without significant co-morbidity and no apparent cardiovascular risk factors presented to us for a second opinion following an emergent admission to her local hospital department with sudden onset central chest pain and ECG changes indicative of acute anterior myocardial infarction (AMI). After initial supportive medical therapy and improvement of her acute symptoms, investigations to further characterise the nature of her coronary disease were performed. CT coronary angiography showed the culprit lesion was likely to be an embolic occlusion of the distal left anterior descending (LAD) artery after the second diagonal branch, but incidentally made note of a soft tissue mass seen on the left coronary cusp of the aortic valve (AV) (Figure 1). Further characterisation of this lesion by both trans-thoracic (TTE) (Figure 2) and trans-oesophageal echo (TOE) showed appearances consistent with a papillary fibroelastoma (PFE) and she was admitted for surgery on the 24th October 2011. A minimal access 6 cm skin incision was made, and the chest entered via a limited upper sternotomy extending to the right 3rd intercostal space. Cannnulation was performed via the ascending aorta and right atrium (Figure 3). Following transverse aortotomy, the aortic valve was inspected revealing a round mass at the very edge of the left coronary cusp. The lesion was excised, sparing the AV, and the valve tested confirming perfect competence prior to closure. Total cross clamp time was 16 minutes with an uneventful wean from bypass. Prior to decannulation, intra-operative TOE confirmed normal valve competence and good overall myocardial function. Total operative time was 52 minutes. The excised lesion measured 9 × 8 × 3 mm (Figure 4). Histopathology demonstrated a fronded papillary lesion, with cores of collagen surrounded by elastic tissue covered with a layer of endocardial endothelium, typical of PFE (Figure 5). The patient had an uneventful post-operative course and was discharged home 3 days after surgery.

## Conclusions

The high risk of thromboembolic complications makes urgent surgical intervention a requirement in the majority of PFEs. Whilst symptomatic patients presenting with non-mobile lesions may be treated with long-term anticoagulation and regular follow up, there is little data reporting outcomes in such patients [3]. Median sternotomy is still the most widely used approach, however, in the absence of other pathology requiring open cardiac surgical intervention, there is increasing evidence that minimally invasive techniques offer excellent exposure and allow for safe resection, particularly in aortic PFEs.

Minimally invasive surgery boasts a number of potential advantages including improved cosmesis, a reduction in post-operative pain, blood loss, subsequent transfusion and shorter intensive care unit and overall hospital stay [27]. Consequently, such techniques may not only lead to greater overall patient satisfaction but also improve hospital resource utilisation. Conversely however, minimally invasive surgery is associated with a steep operator learning curve and, in the case of surgical robotics, the need for costly equipment may limit its uptake in smaller centres. Furthermore, evidence on the optimal surgical approach to PFE also remains limited, and it is unlikely that a randomized controlled trial may ever be performed in light of the low incidence of these tumours.

Our case demonstrates the safe application of a minimally invasive approach to aortic valve papillary fibroelastoma without compromising cross clamp or operative time and achieving an excellent early clinical result. We also present increasing evidence to support the use of minimally invasive approaches in the treatment of PFE, particularly in the aortic position. We feel that in selected patients with PFE minimally invasive surgery should be the approach of choice, and where this is not available, we advocate prompt referral to a specialist centre with the provision and expertise to undertake a minimal access approach.

## Consent

Written informed consent was obtained from the patient for publication of this case report and any accompanying images. A copy of the written consent is available for review by the Editor-in-Chief of this journal.

## Abbreviations

PFE: Papillary fibroelastoma; CT: Computerised tomography; ECG: Electrocardiograph; AMI: Acute mycocardial infarction; TOE: Trans-oespohageal echocardiography; TTE: Trans-thoracic echocardiography.

## Competing interests

There are no financial or non-financial competing interests.

## Author contributions

RC and TA carried out the surgical procedure, participated in provision of clinical information and reviewed the manuscript. VB provided anaesthetic support and trans-oesophageal echo in this case. AN consulted with the patient and provided cardiology care. LH, HA and JK performed the literature review and drafted the manuscript. All authors read and approved the final manuscript.
